# C-Phycocyanin-a novel protein from *Spirulina platensis*- *In vivo* toxicity, antioxidant and immunomodulatory studies

**DOI:** 10.1016/j.sjbs.2020.12.037

**Published:** 2020-12-30

**Authors:** Priyanka Grover, Aseem Bhatnagar, Neeraj Kumari, Ananth Narayan Bhatt, Dhruv Kumar Nishad, Jubilee Purkayastha

**Affiliations:** Institute of Nuclear Medicine and Allied Sciences (INMAS), Defence Research and Development Organisation (DRDO), Brig. S.K. Majumdar Marg, Delhi 110054, India

**Keywords:** C-Phycocyanin, *In Vivo*-toxicity, Antioxidant, Immunomodulatory activities, EDTA, Ethylenediaminetetraacetic acid, SOD, Superoxide Dismutase, GM-CSF, Granulocyte-Macrophage Colony Stimulating Factor, TNFα, Tumor Necrosis Factor α, IL1α, Interlukin 1α, IL1β, Interlukin 1 β, IL2, Interlukin 2, IL4, Interlukin 4, IL6, Interlukin 6, IL10, Interlukin 10, IL12, Interlukin 12, IL13, Interlukin 13, IFN-γ, interferon γ

## Abstract

A pigment-protein highly dominant in Spirulina is known as C-Phycocyanin. Earlier, *in vitro* studies has shown that C-phycocyanin is having many biological activities like antioxidant and anti-inflammatory activities, antiplatelet, hepatoprotective, and cholesterol-lowering properties. Interestingly, there are scanty *in vivo* experimental findings on the immunomodulatory and antioxidant effects of C-phycocyanin. This work is aimed at *in vivo* evaluation of the effects of C-phycocyanin on immunomodulation and antioxidant potential in Balb/c mice. Our results of *in vivo* toxicity, immunomodulatory and antioxidant effects of C-Phycocyanin suggests that C-phycocyanin is very safe for consumption and having substantial antioxidant potential and also possess immunomodulatory activities in Balb/c mice in a dosage dependent manner. C-phycocyanin doesn’t cause acute and subacute toxicity in the animal model (male, Balb/c mice) studied. We have reported that C-phycocyanin exhibited *in vivo* immunomodulation performance in this animal model.

## 1 Introduction

Use of immunomodulatory agents are becoming popular in the management of diseases like cancer, AIDS and autoimmune or inflammatory diseases. However, it also true that the allopathic immunostimulants and immunosuppressants are not free from many limitations associated with them. For example, immunostimulants like levamisole and tetramisole have side effects like skin toxicity and agranulocytosis. Similarly, immunosuppressants like cyclophosphamide, cyclosporine and azathioprine are reported to have side effects like renal toxicity, hepatic toxicity and bone marrow suppression etc. Unlike allopathic immunomdulators, the available natural immunomodulatory products have a greater chance of variation in the active constituents present when crude extracts are used, that minimizes overall therapeutic properties. This limitation can be reduced by the use of pure fractions and extracts (Nandini et al., 2016). Exploring natural products based immunomodulators is growing as an area of great interest and may prove to be very important one for preventing occurrence of various infectious diseases.

Almost all the metabolic disorder is linked with symptoms linked with inflammation and oxidative stress (Peluso et al., 2014). Recently, it has been established that the gutmicrobiota plays an significant role in controlling the physical and physiological conditions and thus plays important role in manifestation of metabolic syndromes in humans. The gutmicrobiota has been an effective target for nutraceuticals. In this context, its worth mentioning that *Spirulina* sp. have been reported to improve the growth of probiotics and also for *in vitro* antimicrobial activity (Finamore et al., 2014).

Many functional food items and beauty products are based on algae (Chen et al., 2014). Spirulina, belonging to the blue green algal group is used as a food supplement because of high-content of protein. Vitamins, minerals and carotenoids are also present in Spirulina in sufficient quantities (Ichimura et al., 2001). Moreover, Spirulina sp has been reported to be effective against hypercholesterolemia, oxidative stress, hyperglycemia etc. and also possesses antihypertensive activity (Torres-Duran et al., 2007).

C-Phycocyanin from Spirulina, a protein, having a brilliant blue color, is composed of two subunits is sold as a colorant for food items as well as for cosmetics (Ichimura et al., 2001). Upto 20% phycocyanin is present in Spirulina protein fraction (Vonshak, 1997, Silveira et al., 2007). It exists as monomers, trimersor hexamers and also as oligomers in small quantities. Phycocyanins include both C-phycocyanin and allophy-cocyanin (Chen et al., 2014).

C-phycocyanin is reported to have many properties (Ores et al., 2016) like antiplatelet (Chiu et al., 2006), hepatoprotective (Ou et al., 2010), antioxidative, anti-inflammatory (Eriksen, 2008, Hirata et al., 2000, Romay et al., 1998a), and cholesterol-lowering properties (Nagaoka et al., 2005).

Hitherto, there are scanty *in vivo* experimental findings on the immunomodulatory and antioxidant effects of C-phycocyanin (Finamore et al., 2014) and further *in vivo* studies are definitely required while going for human studies.

The rationale of the present study is to assess the role of C-phycocyanin on immunomodulation in *in vivo* animal model along with its antioxidant potential.

## 2 Materials and method

### 2.1 Animals and experimental design

The study was conducted according to the OECD guidelines after having approval by the Institutional Ethical Committee, Institute of Nuclear Medicine and Allied Sciences. For the study, six weeks old Balb/c mice (36nos, male) were considered and issued from the animal house facility, Institute of Nuclear Medicine and Allied Sciences (INMAS), Delhi. The animals were acclimatized and allowed to become 8 weeks old before initiating acute and subacute toxicity studies. The animals were given food with standard pellets along with distilled water, kept in plastic cage (n = 3), with sawdust bedding (22 °C, 12 h of day/dark light cycles) (Zulkawi et al., 2017).

### 2.2 Acute toxicity test

As per OECD–420 guidelines, acute oral toxicity test was carried out. Balb/c mice (male) were taken from the INMAS Animal House facility. Six nos of mice were subjected to limit test at 2000 mg/kg BW. Three animals each was given this dose (p.o.) and were observed for 48 h for any toxic symptoms or death. Thereafter observation continued for 14 days. This was followed by three more animals each at the same dose. The LD50 was determined based on OECD guidelines (OECD, 2001).

### 2.3 Sub-acute toxicity test

In subacute toxicity study, phycocyanin was given orally to the mice (30nos Balb/c mice) for a period of 30 days. After acclimatization, the animals were segregated into 5 groups. Control group was given normal diet only along with drinking water (Naidu et al, 2009). The four experimental groups in addition to normal diet and drinking water were given C-phycocyanin at 100, 200, 500, 1000 mg/kg body weight (w/w) of mice. Phycocyanin was obtained from Hash BioTech Labs Private Limited (Chandigarh, India). Food intake was recorded daily and body weights were measured weekly. After the treatment, from each group, three mice were euthanized through carbon dioxide overdose. Blood samples were used for hematological analysis. Serum obtained after centrifugation, was stored at − 80 °C until use for immunological and biochemical analysis. Weights of various organs like brain, heart, liver, lung, kidney, spleen were also noted. The tissues were then embedded in paraffin after fixing into formaldehyde (10%). Histological examination of the tissue sections were done after staining with hematoxylin-eosin (HE) (Lillie, 1965).

### 2.4 Blood Hematological studies

Hematological studies (Gomaa, 2018) of the blood parameters were done using a Horiba Medical analyzer (model MICROS 60, USA).

### 2.5 Preparation of sera samples and serum biochemical analysis

Three mice from each group were euthanized (Moghaddam et al., 2016) through carbon dioxide overdose at fasting state. After blood collection, Serum after separation by centrifugation was stored at − 80 °C until use for immunological and biochemical analyses. Serum Biochemical Analysis was done using Erba Chem-7 (Transasia) Biochemistry Analyzer (Erba, Germany).

### 2.6 Body weight and organs relative weight

After subacute toxicity study, final body weight of the animal in all the groups was noted (Nassef, 2017). Upon being killed liver, kidney, spleen were taken out aseptically and weighed. The relative organ weights were measured as organ weight/final body weight.

### 2.7 *In vivo* antioxidant assessment

The serum antioxidant enzyme activity (Catalase, SOD) levels were compared with normal control and standard control (Vit E). Serum antioxidant enzyme activity (Catalase, SOD) were calculated using kits (Oxiselect, Cell Bio Lab).

Production of superoxide radicals by xanthine and xanthine oxidase leads to the determination of the SOD activity. A red formazon dye is formed when superoxide radical reacts with 2-(4-iodophenyl)-3-(4-nitrophenol)-5-phenyltetrazolium chloride. Briefly, 80 μL of mixed substrate was added to 10 μL of sample, mixed well. Following that, 10 μL xanthine oxidase was added to the reactions and absorbance measured at 490 nm. SOD activity was calculated and expressed as U/L (Oxiselect, Cell Biolabs, Inc.).

Catalase activity (Bahrami et al., 2016) was calculated spectrophotometrically by kit as per manufacturer’s instruction. Briefly, 20 μL of sample was incubated with 50 μL 12 mM of H_2_O_2_ for 1 min. By rapidly adding 50 μL of Catalase Quencher, the reaction was ended. Pink complex of Chromogenic solution and H_2_O_2_ was measured at 520 nm (Park et al., 2005).

### 2.8 Immunomodulatory study by serum cytokines quantification

Twelve nos. of cytokines (viz., IL1α, IL1β, IL2, IL4, IL6, IL10, IL12, IL13, IFN-γ, TNFα, GM-CSF, RANTES) were tested (Pang and Panee, 2014) in the serum after 15 days of dosing of phycocyanin at 100, 200, 500, 1000 mg/kg body weight (w/w) using ELISA kits (Qiagen). The cytokines showing modified expression were recorded and reported.

### 2.9 Statistical analysis

Results were expressed as group mean ± S.E. One-way ANOVA was used for test comparison with controls (p-values < 0.05 considered as significant) and significance assessed with Duncan’s multiple range test.

## 3 Results

### 3.1 Acute toxicity study

Through acute toxicity studies, it was found that the LD50 of the extract was above 2000 mg/kg BW for all the animals used and the drug was safe or non-toxic to mice. There was no mortality or behavioural changes at a dose of 2000 mg/kg, thus indicating a wide margin of the safety of the drug used and there were no observations of obvious toxic symptoms throughout the period of the study. Body weight (BW), organ weight and relative organ weights didn’t show any significant differences (Table 1) when compared between phycocyanin-treated and control mice.

**Table 1:** Body weight, organ weight of normal and 2000 mg/kg C-phycocyanin treated mice for acute toxicity study.

	Normal	C-PC Treated (2000 mg/kg)
Day 0 BW (g)	22.34 ± 0.85	23.15 ± 1.14
Day 14 BW (g)	23.06 ± 0.73	24.21 ± 0.93
Liver weight (g)	1.51 ± 0.08	1.60 ± 0.97
Kidney wt (g)	0.53 ± 0.05	0.55 ± 0.04
Spleen wt. (g)	0.15 ± 0.02	0.22 ± 0.02
Liver/BW ratio	0.065	0.660
Kidney/BW ratio	0.022	0.022
Spleen/BW ratio	0.006	0.009

Values are mean ± SEM of 6 Balb/c mice. No significant difference was observed at p < 0.05.

### 3.2 Sub-acute toxicity study

**Fig. 1:**
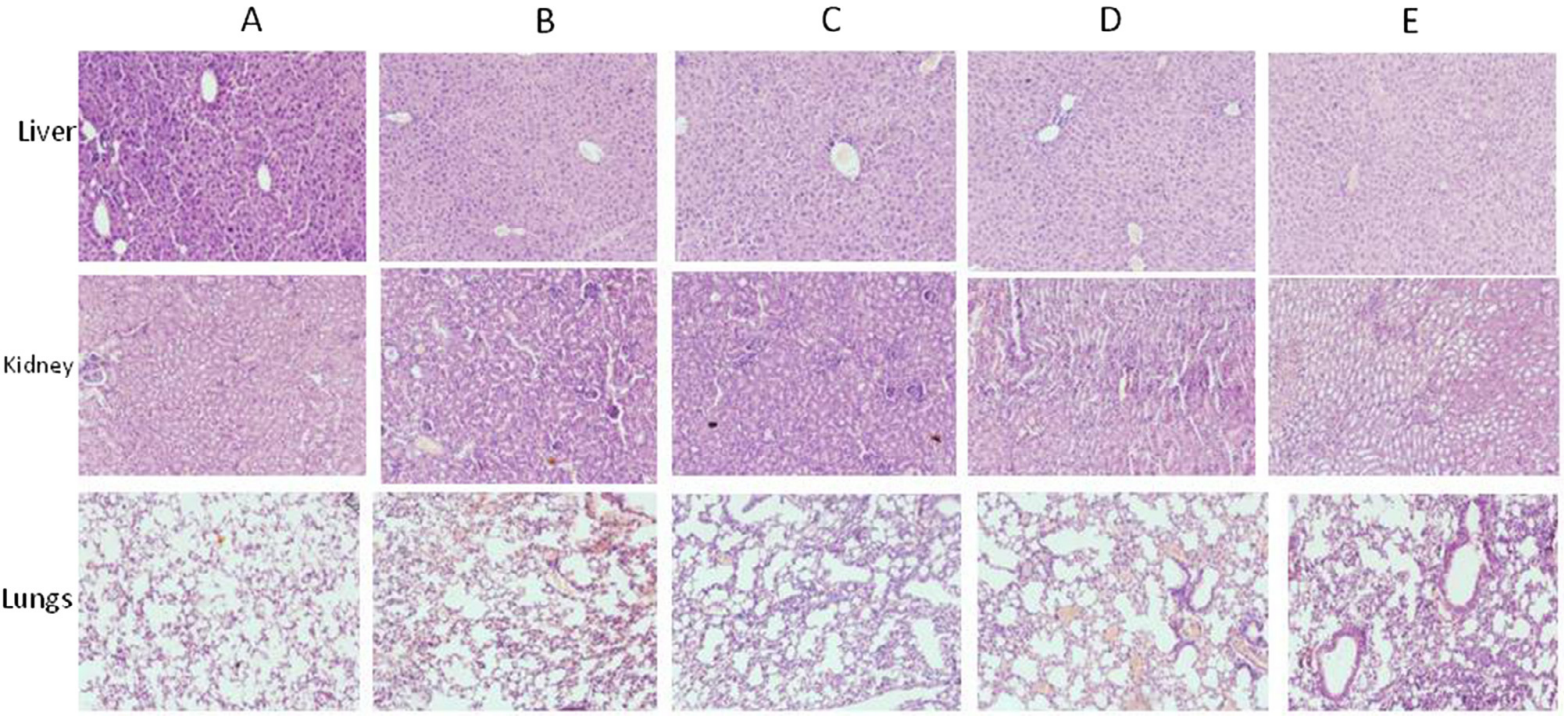
Histological photomicrograph on 31st Day, H & E staining. A. Control group, B. CPC-100 mg/kg group, C. CPC-200 mg/kg group, D. CPC-500 group, E. CPC-1000 mg/kg group.

No significant deviations were found in the body weight and relative organ weights (Table 2) between control and C-phycocyanin treated mice after 30 days of subacute toxicity study. Physical observations didn’t indicate any signs of abnormality like changes in behavior patterns, changes in skin or fur colour, or changes in eyes and mucus membrane. There were no tremors, salivation, diarrhea etc. All the C-PC treated mice survived without significant changes of body or organ weight, sign and symptom of toxicity. Between control and the test groups, significant differences were not observed, so far as clinical observations and biochemical parameters are concerned (Table 3, Table 4, Table 5 and Fig. 1).

**Table 2:** Body weight, organ weight, relative organ weight of normal and 100, 200, 500, 1000 mg/kg body weight of C-phycocyanin treated mice for subacute toxicity study.

	**Normal**	**C-PC (100 mg/kg)**	**C-PC (200 mg/kg)**	**C-PC (500 mg/kg)**	**C-PC (1000 mg/kg)**
Day 0 BW (gm)	22.12 ± 0.78	20.15 ± 0.14	21.02 ± 0.89	21.50 ± 0.69	20.84 ± 0.56
Day 30 BW (gm)	25.34 ± 0.65	23.22 ± 0.78	24.69 ± 0.45	25.21 ± 0.63	23.89 ± 0.79
BW Gain	3.22 ± 0.13	3.07 ± 0.64	3.67 ± 0.44	3.71 ± 0.06	3.05 ± 0.23
Liver weight (gm)	1.41 ± 0.57	1.39 ± 0.98	1.40 ± 0.91	1.45 ± 0.78	1.38 ± 0. 68
Kidney wt (gm)	0.55 ± 0.06	0.51 ± 0.04	0.56 ± 0.02	0.58 ± 0.07	0.49 ± 0.05
Spleen wt. (gm)	0.24 ± 0.07	0.21 ± 0.05	0.27 ± 0.59	0.28 ± 0.70	0.21 ± 0. 06
Liver/BW ratio	0.055	0.059	0.056	0.057	0.057
Kidney/BW ratio	0.021	0.021	0.022	0.023	0.020
Spleen/BW ratio	0.009	0.009	0.010	0.011	0.008

Values are mean ± SEM of 5 Balb/c mice. No significant difference was observed at p < 0.05.

**Table 3:** Hematological values in BALB/c mice treated with different doses of C-phycocyanin in comparision with control.

**Hematology Parameters**	**CPC-C**	**CPC-100**	**CPC-200**	**CPC-500**	**CPC-1000**
Haemoglobin (g/dl)	12.4 ± 1.4	12.8 ± 1.2	14.8 ± 0.9	13.8 ± 1.2	13.8 ± 1.2
TLC (×10^3^/mm^3^)	2.7 ± 0.9	2.5 ± 0.7	2.8 ± 0.5	2.7 ± 0.9	2.7 ± 0.9
R.B.C. Count (×10^6^/mm^3^)	8.2 ± 0.9	8.8 ± 0.9	8.5 ± 1.5	8.7 ± 1.2	8.7 ± 1.2
Haematocrit (%)	42.1 ± 2.8	44.2 ± 2.4	47.2 ± 1.7	46.2 ± 1.5	46.2 ± 1.5
MCV (fL)	47.6 ± 3.6	47.2 ± 2.6	48.2 ± 2.1	49.2 ± 1.1	49.2 ± 1.1
MCH (pg)	14.9 ± 1.0	14.5 ± 1.4	15.1 ± 1.1	16.2 ± 1.2	16.2 ± 1.2
MCHC (%)	32.8 ± 2.5	33.1 ± 1.7	32.1 ± 1.5	33.2 ± 1.4	33.2 ± 1.4
Platelet count (×10^3^/mm^3^)	607 ± 121	657 ± 145	678 ± 130	689 ± 141	689 ± 141

Values are mean ± SEM of 5 Balb/c mice. No significant difference was observed at p < 0.05.

**Table 4:** Differential Leucocyte Count (%) (neutrophils, lymphocytes, eosinophils, monocytes and basophils) in BALB/c mice treated with different doses of C-phycocyanin.

**Differential Leucocyte Count (%)**	**CPC-100 (mg/kg)**	**CPC-200 (mg/kg)**	**CPC-500 (mg/kg)**	**CPC-1000 (mg/kg)**
Neutrophil	22.6 ± 1.7	21.9 ± 1.2	22.9 ± 1.4	21.8 ± 1.4
Lymphocyte	75.2 ± 1.8	75.4 ± 2.4	74.9 ± 1.7	74.9 ± 2.1
Eosinophil	1.2 ± 0.02	0.9 ± 0.05	1.4 ± 0.05	1.1 ± 0.09
Monocyte	3.2 ± 0.5	3.09 ± 0.8	3.7 ± 0.8	3.5 ± 0.4
Basophil	0.1 ± 0.01	0	0.1 ± 0.02	0.1 ± 0.01

Values are mean ± SEM of 5 Balb/c mice. No significant difference was observed at p < 0.05.

**Table 5:** Results of biochemical values of Balb/c Mice treated with different dosage of C-phycocyanin in comparision with control.

Biochemical Parameters	CPC-C	CPC-100	CPC-200	CPC-500	CPC-1000
Bilirubin (mg/dl)	0.80 ± 0.05	0.78 ± 0.07	0.79 ± 0.09	0.82 ± 0.05	0.81 ± 0.07
Total Protein (gm/dl)	4.21 ± 0.41	4.47 ± 0.33	4.32 ± 0.12	4.31 ± 0.12	4.41 ± 0.18
Albumin (gm/dl)	4.11 ± 0.22	3.89 ± 0.13	3.99 ± 0.50	3.10 ± 0.21	3.99 ± 0.42
Globulin (gm/dl)	2.52 ± 0.12	2.51 ± 0.18	2.49 ± 0.19	2.50 ± 0.21	2.51 ± 0.23
A/G Ratio	1.63	1.54	1.60	1.24	1.58
SGOT (U/l)	238.60 ± 1.75	241 ± 1.21	240 ± 1.67	242.60 ± 1.89	239 ± 1.78
SGPT (U/l)	90.31 ± 1.71	90.21 ± 1.67	89.41 ± 1.78	90.11 ± 1.75	88 ± 1.71
Blood Urea (mg/dl)	40.17 ± 1.21	42.11 ± 1.34	41.43 ± 1.32	43.21 ± 1.05	42.78 ± 1.72
Serum Creatinine (mg/dl)	0.50 ± 0.05	0.51 ± 0.07	0.54 ± 0.09	0.55 ± 0.07	0.53 ± 0.08
Total Cholesterol (mg/dl)	85.07 ± 3.51	86.03 ± 2.47	85.12 ± 2.74	87.14 ± 2.98	85.56 ± 2.58
Serum Uric Acid (mg/dl)	2.07 ± 0.04	2.08 ± 0.05	2.09 ± 0.02	2.05 ± 0.05	2.07 ± 0.01
Blood Urea Nitrogen (mg/dl)	21.45 ± 0.82	22.01 ± 0.78	21.89 ± 0.72	21.58 ± 0.75	21.41 ± 0.71

Values are mean ± SEM of 5 Balb/c mice. No significant difference was observed at p < 0.05.

### 3.3 *In vivo* antioxidant assay

**Fig. 2:**
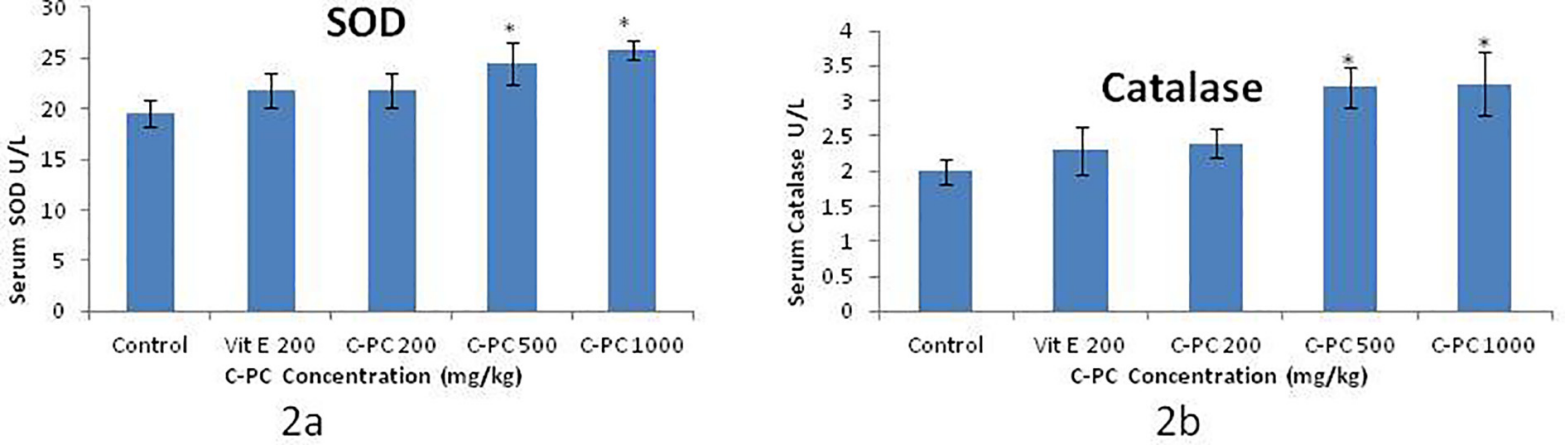
Effect of different concentrations of C-PC on Serum Superoxide Dismutase (SOD) and Catalase activity in comparison to Control and Vit E. Vit E 200: Vitamin E 200 mg/kg; C-PC 200: C-phycocyanin 200 mg/kg; C-PC 500: C-phycocyanin 500 mg/kg; C-PC 1000: C-phycocyanin 1000 mg/kg. *indicates a significant difference compared with Control and Vit E group (*P* < 0.05).

C-phycocyanin at 500 mg/kg and 1000 mg/kg resulted in significant enhancement of serum SOD activity that is higher than that of vitamin E (Fig. 2a) while C-PC at 200 mg/kg has SOD activity compared to Vit E at 200 mg/kg (*P* < 0.05). In a similar manner C-phycocyanin at 500 mg/kg and 1000 mg/kg resulted in significant enhancement of serum catalase activity that is higher than that of vitamin E (Fig. 2b) while C-PC at 200 mg/kg has catalase activity compared to Vit E at 200 mg/kg (*P* < 0.05).

### 3.4 Immunomodulatory study

**Fig. 3:**
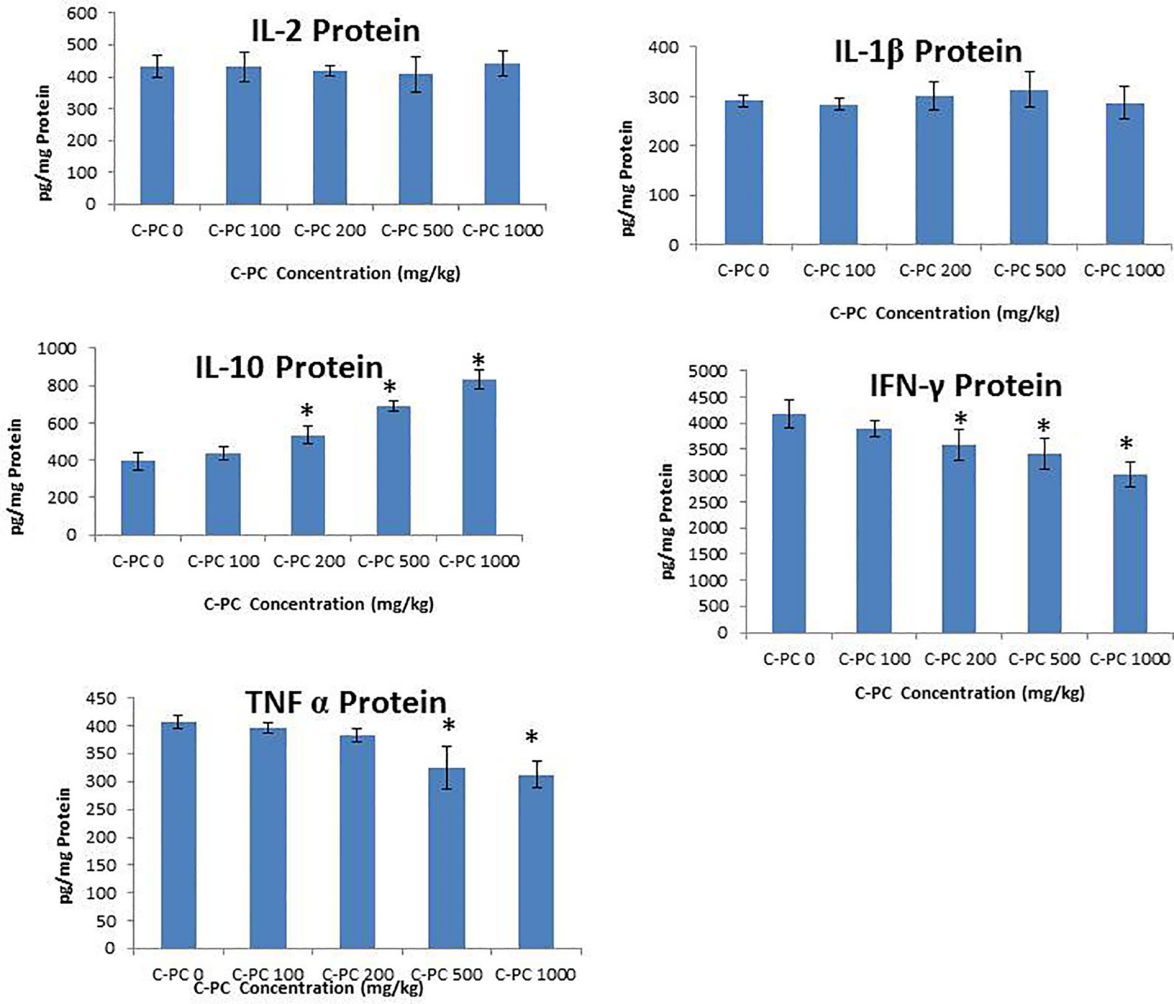
Effect of different concentrations of C-PC on Serum Cytokines levels in comparison to Control. *indicates a significant difference compared with Control and Vit E group (*P* < 0.05).

Serum cytokines levels were also estimated to understand the immunity of the C-PC treated healthy mice. Expression of twelve nos. of cytokines (viz., IL1α, IL1β, IL2, IL4, IL6, IL10, IL12, IL13, IFN-γ, TNFα, GM-CSF, RANTES) in the serum were determined using ELISA kits (Qiagen). It was found that C-phycocyanin suppresses the synthesis of pro-inflammatory cytokines, interferon-γ (IFN-γ), and tumor necrosis factor-α (TNF-α) in a concentration dependent manner (Fig. 3). The levels of TNF-α and IFN-γ were significantly decreased in 500 and 1000 mg/kg treated groups in comparison with controls. The levels of IL-2 and IL-1β were not significantly affected in the C-Phycocyanin treated groups. However, C-phycocyanin enhances the levels of anti-inflammatory cytokines, such as IL-10 in a concentration-dependent manner (Fig. 3).

## 4 Discussion

C-Phycocyanin has a wide margin of safety as there was no mortality or behavioural changes at a dose of 2000 mg/kg, and there were no observations of obvious toxic symptoms throughout the period of the study. No significant deviations were found in the body weight between control and C-phycocyanin treated mice after 30 days of subacute toxicity study.

Also, after the treatment period, the C-PC treated mice didn’t show any signs and symptoms of toxicity. The clinical and biochemical parameters study as well as histopathological evaluations of the kidney and liver revealed normal status. Thus, the results of the present study showed that C-phycocyanin from *Spirulina platensis* did not bring on any detrimental effects in Balb/c mice. These findings provides sufficient evidence to conclude that the orally administered C-phycocyanin was safe and showed no toxicity even at the maximum dose of 2,000 mg C-phycocyanin per Kilogram of body weight.

C-phycocyanin, is gaining popularity because of their many bioactivities already reported from time to time. C-phycocyanin has been known for anti-tumor, antioxidant and anti-inflammatory properties based on few earlier studies (Strasky et al., 2013, Eriksen, 2008; Bhat and Madyastha, 2000; Romay et al., 1998a, Romay et al., 1998b, Romay et al., 2000) and is considered as a raw material for making various nutraceutical products (Silveira et al., 2007). Even anticancer bioactivity has been attributed to C-phycocyanin (Li et al., 2010, Wang et al., 2007).

Also, C-phycocyanin was able to lessen the production of macrophages (RAW 264.7) with growing dosages (Reddy et al., 2003). C-phycocyanin was able to particularly curb COX-2 and PGE2 expression when RAW 264.7 macrophages were activated by lipopolysaccharides (LPS), thus, establishing the anti-inflammatory nature of C-phycocyanin. Oxidative stress plays significant roles in processes of ageing and pathogenesis of numerous diseases like diabetes, cancer, neurodegenerative and respiratory tract disorders (Anderson et al., 2000). Halliwell et al. (1996) opined that the sum of endogenous and food derived antioxidants correspond to the total antioxidant capability of a system. The role of antioxidant is to detoxify reactive oxygen intermediates in the body (Delay, 1993). Therefore, improved antioxidant status can minimize oxidative stress and associated damages. This delays or decreases the risk of developing free radical induced diseases. Protective antioxidants bestowed by many plant extracts and products make these agents promising therapeutic drugs for free radicals induced pathologies. *In vitro* antioxidant properties of C-phycocyanin has been demonstrated earlier (Romay et al., 1998b). Similarly, Chen et al., (2014) has shown that LPS stimulation of J774A.1 macrophages rapidly stimulate ROS production in comparision with control cells. LPS-induced ROS was reduced when pretreatment was done with N-acetylcysteine (NAc). The ROS (H_2_O_2_) content decreased within 2 h when C-phycocyanin was present. .

In this study, C-phycocyanin at 500 mg/kg and 1000 mg/kg resulted in significant (*P* < 0.05) enhancement of serum SOD and catalase activity that is higher than that of vitamin E while at 200 mg/kg C-PC has SOD and catalase activity (Sharma and Mahajan, 2013) compared to that of Vit E (*P* < 0.05).

CAT, SOD and GSH-Px enzymes are known to be very important scavenger of hydrogen peroxide as well as of superoxide ion. These enzymes play vital role in shielding the cellular constituents from oxidative damage (Scott et al., 1991). Superoxide dismutase (SOD) is considered as an enzyme widely used as a biochemical marker of disease condition caused by oxidative stress (Dündarz et al., 2003). Many harmful oxidative changes are associated with decrease in the levels of CAT, SOD.

C-phycocyanin in our studies has shown to have added to the SOD and CAT activities and thus in turn has a higher free radical absorbing capacity. Thus, C-phycocyanin can have a beneficial action against the harms caused by O2^•^ and OH^•^.

Moreover, due to toxicity of some of the synthetic drugs, there is high demand for the natural immunomodulators. Immunomodulation by herbal way is the most acceptable one owing to the demerits of allopathic immunomodulators. The worldwide demands for the natural immunomodulators are difficult to meet and many biotechnological methods are also under development.

Cytokines plays crucial role in regulating the immune response. Under natural conditions, pro-inflammatory cytokines plays important role in the development of suitable defence system. In first-line of immunological defense in mammals, macrophages clean out tumor cells (Park et al., 2009) through the release of diverse cytokines (Adams and Hamilton, 1984). For example, TNF-α has cytotoxic effects (Bowdish et al., 2007, Striz et al., 2014, Biswas and Mantovani, 2014). IFN-α, promotes type 1 immune responses and obstruct the growth of cancer cells (Werneck et al., 2008, Lee et al., 2011). IL-2 controls the functions of white blood cells. IL-1β enhances the production of T-cells, stimulate B-cells along with few other functions. The beginning of the innate immune response is mainly by inflammatory cytokines like TNF-α, IL-1β and IL-6 and also plays important role in determining the extent of acquired immune response (Netea et al., 2003). Earlier *in vitro* studies have shown that, C-PC possess antioxidant as well as immunomodulatory performance (Chen et al., 2014). In our study, the immunity of the C-PC dosed healthy mice was detected by estimating the quantities of various cytokines in the serum. Expression of twelve nos. of cytokines (viz., IL1α, IL1β, IL2, IL4, IL6, IL10, IL12, IL13, IFN-γ, TNFα, GM-CSF, RANTES) in the serum were determined using ELISA kits (Qiagen). It was found that C-phycocyanin suppresses the synthesis of pro-inflammatory cytokines, interferon-γ (IFN-γ), and tumor necrosis factor-α (TNF-α) in a concentration dependent manner (Fig. 3). The levels of TNF-α and IFN-γ were significantly decreased in 500 and 1000 mg/kg treated groups in comparison with controls. The levels of IL-2 and IL-1β were not significantly affected in the C-Phycocyanin treated groups. However, C-phycocyanin enhances the levels of anti-inflammatory cytokines, such as IL-10 in a concentration-dependent manner (Fig. 3). Therefore, our study concludes that C-phycocyanin suppresses the production of TNF-α and IFN-γ without putting any inhibitory effect on the production of anti-inflammatory cytokines like IL 10.

## 5 Conclusion

In conclusion, our results of *in vivo* toxicity, immunomodulatory and antioxidant effects of C-Phycocyanin confirms that C-phycocyanin is very safe for consumption as it doesn’t cause acute and subchronic toxicity. Moreover, we found that C-phycocyanin strengthens immunity as well as have a very potent effect on serum antioxidant level. It may have the potential to be considered as an important nutraceutical supplement to get rid of various infectious as well as oxidative stress induced diseases.

## Declaration of Competing Interest

None.
